# Audiohaptic Feedback Enhances Motor Performance in a Low-Fidelity Simulated Drilling Task

**DOI:** 10.3390/brainsci10010021

**Published:** 2019-12-31

**Authors:** Brianna L. Grant, Paul C. Yielder, Tracey A. Patrick, Bill Kapralos, Michael Williams-Bell, Bernadette A. Murphy

**Affiliations:** 1 Faculty of Health Sciences, Ontario Tech University, Oshawa, ON L1G 0C5, Canada; Brianna.grant@ontariotechu.net (B.L.G.); paul.yielder@uoit.ca (P.C.Y.); Tracey.Patrick@uoit.ca (T.A.P.); 2 Faculty of Business and Information Technology, Ontario Tech University, Oshawa, ON L1G 0C5, Canada; Bill.Kapralos@uoit.ca; 3 Durham College, School of Health and Community Services, Oshawa, ON L1G 0C5, Canada; Michael.Williams-Bell@durhamcollege.ca

**Keywords:** simulation, audiohaptic, multisensory perception, motor control, body representation

## Abstract

When used in educational settings, simulations utilizing virtual reality (VR) technologies can reduce training costs while providing a safe and effective learning environment. Tasks can be easily modified to maximize learning objectives of different levels of trainees (e.g., novice, intermediate, expert), and can be repeated for the development of psychomotor skills. VR offers a multisensory experience, providing visual, auditory, and haptic sensations with varying levels of fidelity. While simulating visual and auditory stimuli is relatively easy and cost-effective, similar representations of haptic sensation still require further development. Evidence suggests that mixing high- and low-fidelity realistic sensations (e.g., audition and haptic) can improve the overall perception of realism, however, whether this also leads to improved performance has not been examined. The current study examined whether audiohaptic stimuli presented in a virtual drilling task can lead to improved motor performance and subjective realism, compared to auditory stimuli alone. Right-handed participants (*n* = 16) completed 100 drilling trials of each stimulus type. Performance measures indicated that participants overshot the target during auditory trials, and undershot the target during audiohaptic trials. Undershooting is thought to be indicative of improved performance, optimizing both time and energy requirements.

## 1. Introduction

Virtual reality, computer-based simulations, and live simulations, collectively referred to as immersive simulated environments (ISEs), have the unique ability to replicate scenarios in diverse contexts, including those pertaining to the acquisition, development, and maintenance of cognitive and psychomotor skills [1]. ISEs provide a safe, cost-effective alternative to real world environments, which may present challenges that make obtaining adequate practice difficult. Users have the ability to practice rare (and sometimes life-threatening) events in a controlled manner in an ISE, while researchers analyze the user’s performance and provide feedback, such as accuracy and response time [2,3]. Users can perform multiple iterations of a task with relevant feedback to develop proficiency before taking their skills to the real world, where psychomotor errors can result in serious consequences. Practicing new motor skills is essential to optimize performance [4], however, it is not clear how various sensory percepts experienced during training impact skill acquisition and learning.

ISEs can be useful for rehabilitation and training purposes, especially within high-stress occupations, such as medical professionals, firefighters, and military personnel [2,3,5,6]. When individuals with these occupations make performance errors, it is likely at the expense of individual or public safety. It is essential that we understand how to reduce the risk of these errors, and it is possible that repeated exposure to realistic scenarios can enhance the user’s cognitive and psychomotor behavior while performing tasks. Computer graphics technology has greatly enhanced visual immersion by providing realistic renderings of computer-generated worlds, while other sensory feedback, such as audition and touch, have seen growing research with recent advances in consumer-level technologies [7].

As interactive multimedia technology advances and virtual worlds become more realistic, there is an increasing need to expand the understanding of how ISEs impact human brain function and task performance. In a real environment, multiple sensory modalities are constantly being stimulated and are providing crucial information to be organized and integrated through multisensory integration [8]. However, little research has been done to consider how haptic sensation may alter the other senses, despite the importance of touch in our daily lives. Haptic perception refers to sensation generated through touch and manipulation, which includes both tactile and kinesthetic stimuli [9,10]. Without haptic perception, humans would have great difficulty discriminating textures between multiple surfaces, and would struggle to grasp and manipulate objects for tool use. The field of haptics largely consists of information acquisition by machines, humans, or a combination of the two, in real or simulated environments [9,11]. Simulating this sense can be difficult, as it requires realistic sensation of pressure, texture, movement, and vibration, which are constrained by inherent limitations of diverse actuators [10,11].

Low-end haptic devices are readily available to consumers and are relatively inexpensive, however, they may not replicate real-world operations as closely as their high-end counterparts in terms of force feedback, resolution, and refresh rate [11]. Low-fidelity haptics provide simple vibration stimuli from motors, and are found in devices such as video-game controllers, portable gaming consoles, haptic gloves, and mobile devices. Other low-end haptic devices employ serial, parallel, and other mechanisms to provide force and tactile feedback [11]. In order to accurately replicate real-world tasks, these devices often need higher levels of fidelity and range of motion (degrees-of-freedom), which lower-end devices fail to provide [12]. Devices with high levels of haptic fidelity can be costly and are often utilized in tasks requiring precision, such as in teleoperations, delicate surgical procedures using robotics, and when astronauts operate robots for external repairs on the International Space Station [10]. Research has shown the importance of high-end haptics in the medical field, where advanced computer simulations allow student practitioners to train on virtual (simulated) patients while receiving feedback on their performance [3,13]. Haptics in the form of force-feedback is often used to replicate the mechanical properties of individual organs, tissues, or entire cadavers [3,14]. High-end haptic devices can certainly provide more tactile information regarding surface properties; however, access may be limited due to the costs associated with maintenance, infrastructure, and acquisition for large groups of trainees. It is still uncertain whether the increased haptic fidelity in high-end devices are beneficial in regards to enhanced motor performance and learning [11]. If low-fidelity haptic devices utilized in an ISE can achieve high levels of performance and retention, while enhancing the simulation experience, then they may be useful devices for training-based simulations.

Research suggests that simultaneous exposure to multiple sensory modalities can influence a user’s perception, and interactions between congruent stimuli can be beneficial for performance [11,15]. Perception of our own body in space involves the coordination of proprioceptive and kinesthetic information, which is fundamental for interacting with the world around us [16,17]. Awareness of joint position allows us to sustain posture and execute movements efficiently [17], and is classically known as body schema [18]. Research suggests that the primate brain constructs internal body-centered representations that can be modified following a period of tool-use, which extends the reachable space near the user (peripersonal space) [19,20]. With prolonged use, a tool can become an extension of the hand in regards to our internal body representation [16]. In theory, this should also apply to objects or machinery that a user regularly interacts with. The plasticity of our body schema is essential for the manipulation of objects to gather information from our environment. Simulation research provides opportunities for trainees to practice with a variety of tools in a controlled environment, ultimately obtaining skill proficiency that is ideally transferred to the real scenario.

A recent emergence of haptic and audiohaptic interactions within simulation research more accurately replicates the multimodal environment in which humans actually perform [21,22]. We are constantly engaging with various stimuli, and when two or more sensory inputs are congruent and presented simultaneously, we are able to process and respond to our environment more quickly than if the stimuli were presented separately [15,23]. Visual and auditory interactions are among the most studied, however, there is an increase in research investigating the role of technology in human–haptic interactions [3,10,11]. Sound can induce an intimate response to our physiology, as discussed in a recent study that found an increase in cortisol levels during video game play with music, as compared to the same game without music [24]. Manipulating auditory stimuli in video games can also influence a user’s play experience, which ultimately affects their feelings of presence [11,25]. Presence refers to the feeling of being situated within a virtual environment regardless of the user’s physical location, and is thought to be conducive to effective performance in the virtual world [26]. This experience can be challenging to measure, and questionnaire-based approaches are inherently biased, in that asking about presence may induce the very feeling the questionnaire is trying to measure [26]. Behavioral observations, physiological measurements, and correlates of neural activity have all been utilized as an attempt to objectively measure the presence experience [26,27,28].

Some research suggests that the sense of touch provides direct features from the environment, including the physical properties of the surface of manipulated objects, while visual stimuli provides ambiguous sensory information about physical properties [29]. Visual dominance, however, is a well-known phenomenon that explains how visual stimuli typically dominate other percepts (i.e., audition, proprioception, etc.) and drives the multimodal interaction [30,31]. Hartcher-O’Brien and colleagues [32] conducted a series of studies to determine if vision also dominates the sense of touch. They found that during a speeded discrimination task, participants made more visual-only responses than tactile-only responses in bimodal trials [32]. It is clear that vision plays a critical role in responses that require speed, however, most of these studies use simple response paradigms, where the haptic information provided is not task-relevant. Lin and colleagues [33] found that participants made fewer errors when receiving haptic feedback in a visuomotor tracking task, as compared to not receiving haptic feedback. However, their study included a haptic joystick as the haptic interface, which may not be relevant to tasks performed in a real environment. These findings indicate that greater research is required to explore how task-relevant haptic feedback can influence motor learning adaptations and task performance in simulations and virtual environments.

The current study uses both auditory stimuli and task-relevant audiohaptic stimuli to explore the role of low-fidelity haptic sensation on motor performance and the user’s perceived ratings of reality. Our research objective was to examine how low-fidelity auditory and haptic sensations affect performance and perceived realism during a simulated drilling task. Our hypotheses were that force-feedback presented in a virtual wood-drilling simulation would increase ratings of perceived realness and enhance user performance.

## 2. Materials and Methods

### 2.1. Participants

This research was carried out in accordance with the Declaration of Helsinki, and the protocol was approved by Ontario Tech University’s Research Ethics Board (reference #15042). All participants provided written informed consent prior to participating in the study. Participants were volunteers recruited from the student population at Ontario Tech University, in Oshawa, Canada. Recruitment was done through in-course announcements and by word of mouth. Participants recruited were young adults between the ages of 18–35 years old that were right-hand dominant and self-reported normal vision and hearing. The mean age of participants was 22.9 ± 1.4 years old (*n* = 16, seven females). All participants reported prior experience using a drill: eleven participants reported minimal prior experience, four participants reported moderate experience, and one participant reported frequent drill use at a job he worked at several years prior. An a priori sample size calculation revealed 15 participants were needed to obtain a large effect size, α-value of 0.05, and power of 0.8 [34].

The Edinburgh Handedness Questionnaire was used to assess participants’ hand dominance, with results indicating right-handed, left-handed, or ambidextrous. Fifteen participants reported right-handedness and one participant reported being ambidextrous. Handedness was measured to ensure participants could effectively operate the haptic controller, which was placed to the right of the computer screen that displayed the simulation.

### 2.2. Auditory Stimuli

The drilling sounds used in the simulation were actual recordings from a Stanley Black and Decker (New Britain, CT, USA) consumer drill, drilling in open air and drilling through wood. The recordings were made in an audiometric room to limit external noise and reverberation of sounds within the environment [11]. Two separate auditory stimuli were used in every trial; an initial “air” recording, representing a drill operating in open air, followed by a “wood” audio clip, recorded from the sounds made from drilling through a block of wood. The air recording was presented with the start of the simulation and stopped once the drill bit made contact with the virtual block of wood, at which point the wood recording started. The wood recording was looped to play throughout the drilling movement. These two recordings allowed the auditory stimuli to be synchronized with the movements of the user, however, they did not change with the depth of the material, nor with pressure changes from the user.

All auditory stimuli were played through external Sony (Tokyo, Japan) speakers, adjacent to a 23-inch display monitor (LG Corporation, Busan, South Korea) depicting the simulation. Auditory stimuli were presented at the same volume for all participants.

### 2.3. Audiohaptic Stimuli

**Figure 1:**
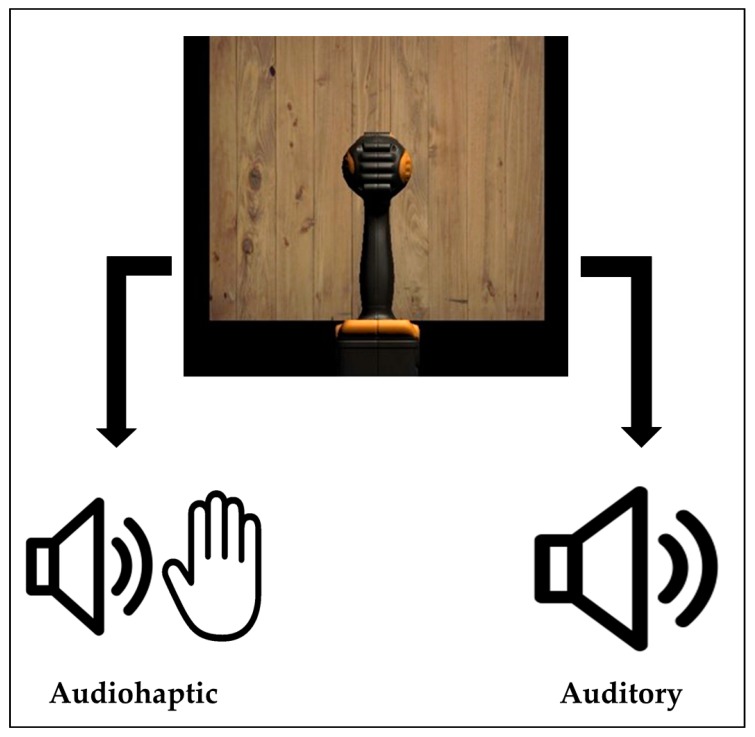
Stimulus conditions presented in each trial type (audiohaptic or auditory alone).

The Novint Falcon, a low-fidelity haptic device by Novint Technologies (Albuquerque, NM, USA), was used to provide haptic force-feedback in the audiohaptic trials. The Falcon provides up to 9.0 N of force-feedback with three degrees of freedom, with a resolution of 400 dpi (dots per inch). Higher-fidelity haptic devices can typically provide up to 40 N of force, with five to seven degrees of freedom [3]. As described in [11], the haptic sensations provided in this simulation were modeled after the resulting forces and vibrations measured from a real drill drilling through a piece of wood. Haptic forces were simulated using a spring-mass system coded into the Unity game engine (Unity Technologies, San Francisco, CA, USA). A lightweight 3D-printed mock drill was used as the haptic interface to provide realistic tactile input. When a participant pushed the drill forwards to contact the virtual block of wood during an audiohaptic trial, the Falcon provided resistance to the user’s motion. The haptic stimuli and wood audio recording occurred simultaneously. Figure 1 displays the stimuli presented in both trial types.

### 2.4. Procedures

A computer-generated drilling task was utilized to assess motor behavior in a simulated environment. The task was to drill two centimeters (2 cm) into a virtual block of wood, and then rate perceived levels of realism after completion of each trial. The simulation was created using Unity game engine (Unity Technologies, San Francisco, CA, USA), and has been utilized in previous simulation research at Ontario Tech University [11]. Stimuli were presented in four blocks, with each block consisting of 50 trials (25 per condition). The first two blocks (100 trials) were randomized and then repeated, for a total of 200 trials. Five-minute rest periods were allotted every 50 trials, or upon the participants’ request. Participants were seated facing the display monitor, with their eyes positioned approximately 72 ± 5 cm away from the screen (measured with a measuring tape). The base of the Novint Falcon was fixed to the table on the right side of the display monitor, while the three moveable arms of the device allowed participants to easily grasp and maneuver the 3D printed drill handle to complete the task.

**Figure 2:**
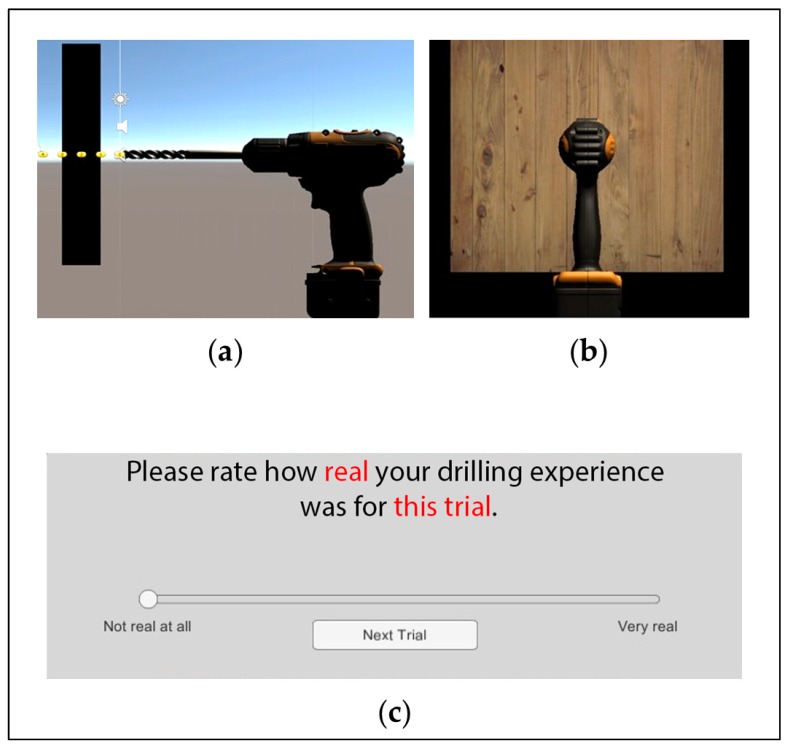
Scenes in the drilling simulation. (**a**) Side view visible only during familiarization trials. (**b**) Front view shown throughout experimental trials. (**c**) Subjective rating scale presented after each trial.

A five-minute familiarization phase was implemented before the experiment began to ensure participants felt confident in operating the Falcon to complete the task. During this phase, participants were able to view a lateral aspect of the simulation (see Figure 2a). This side-view provided immediate visual feedback on the depth of the drill-bit as the participant moved the Falcon forward to contact the virtual block of wood. Once participants verbally indicated they were confident with operating the Falcon, they began the experimental phase. A front-view of the simulation, Figure 2b, was the only visual stimuli presented to participants throughout the 200 trials of this phase. The lack of visual information presented during the experimental trials was utilized to isolate the sensation of haptic feedback, and may provide a more realistic scenario (i.e., drilling a hole into drywall, where there is a lack of visual feedback).

After completion of each trial, participants were asked to rate how realistic the drilling experience was. This was done using a continuous linear visual analogue scale, with “not real at all” representing a score of zero (0), and “very real” representing a score of ten (10). This type of rating scale was used to deter participants from memorizing previous ratings. The focus of our study was to examine whether low-fidelity haptic sensations had the capacity to enhance performance. Due to the challenges in measuring sense of presence discussed previously [26,27], we only asked participants about the sensory aspects of the simulated task. Therefore, after each trial, participants were asked to rate their perceived realness of the sensory stimuli presented in that trial. A screenshot of the subjective rating scale can be seen in Figure 2c. For each trial, drilled depth and rating of perceived realness were recorded.

### 2.5. Data Acquisition and Analysis

#### 2.5.1. Performance Data

The Unity game engine (Unity Technologies, San Francisco, CA, USA) was used to run the simulation, and collect and record performance data. Performance measures were obtained from all participants by recording mean drilled depth and calculating absolute, constant, and variable errors for both trial conditions (see Supplementary Tables S1 and S2). Absolute error represents the average error without reference to direction, whereas constant error indicates the direction of error, and variable error refers to the variability of performance trials [35,36,37]. Absolute error was calculated as the absolute value of the difference of the drilled depth and the target depth (2 cm; Equation (1)), constant error was calculated as the signed difference between drilled depth and target depth (Equation (2)), and standard deviation of constant error was used as variable error (Equation (3)).

Absolute Error = |drilled depth − target depth|,(1)

Constant Error = drilled depth − target depth,(2)

(3) $$\mathrm{Variable}\;\mathrm{Error}=\sqrt{\frac{\Sigma {\left(x-\bar{x}\right)}^{2}}{n}}.$$

#### 2.5.2. Perceived Realness Data

Subjective ratings of reality were obtained after every trial, with participants clicking and dragging a computer mouse to a desired location along the linear scale to indicate their rating. Mean ratings were recorded for each trial condition (see Supplementary Tables S1 and S2).

### 2.6. Statistical Analysis

Mean drilled depth, absolute error, constant error, and variable error were calculated for each participant in response to each trial type. The mean rating of realness was also calculated for both conditions for every participant. Data points beyond ±2 standard deviations were removed in each dependent variable (drilled depth, absolute error, constant error, and variable error) prior to statistical testing, which accounted for 6% of all data. A 3 performance measure (absolute, constant, and variable error) by 2 stimulus (audiohaptic and auditory alone) multivariate analysis of variance (MANOVA), with repeated measures on the last factor, was conducted to determine significant differences in task performance. To determine differences in biological sex, a 3 performance measure by 2 sex (male and female) by 2 stimulus MANOVA, with repeated measures on the last factor, was conducted. Subjective ratings of reality were compared between auditory and audiohaptic trials using a paired samples *t*-test. Alpha was set as *p* < 0.05, and partial eta squared (*η*p^2^) values were reported for estimates of effect size, where 0.01 indicated a small effect size, 0.06 a medium effect size, and 0.14 a large effect size [38]. All statistical tests were run using SPSS^®^ version 26 (Armonk, NY, USA). All data was checked for normality using Shapiro–Wilk’s test.

## 3. Results 

### 3.1. Performance Results

**Figure 3:**
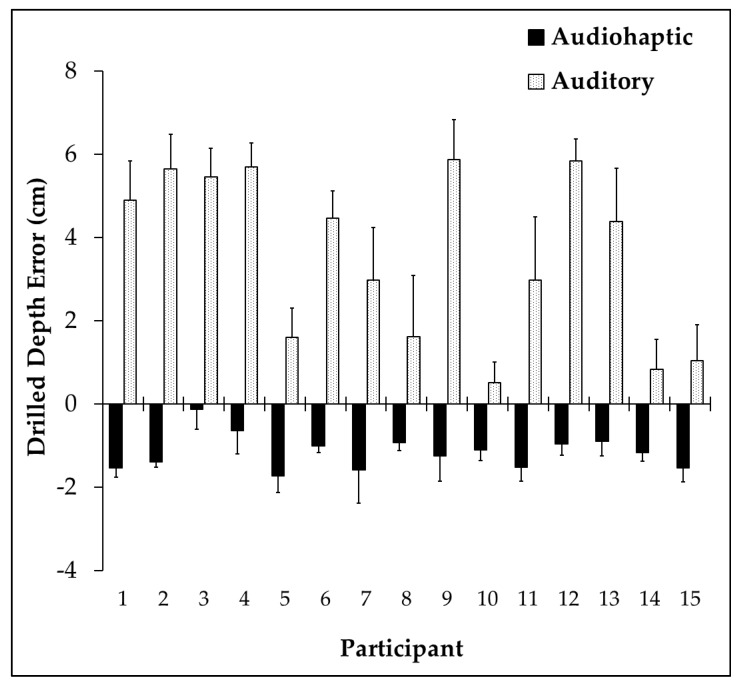
Mean and standard deviation (error bars) of participants’ drilling behavior for each trial type.

Means and standard deviations are critical for statistical analysis, however it is equally important and perhaps more interesting to consider how each individual participant behaved when exposed to the different stimulus types. Figure 3 demonstrates the drilling behaviors (constant error) of each participant in both audiohaptic and auditory-only conditions. Participants 1–10 reported minimal past experience with drills, participants 11–14 reported moderate experience, while participant 15 reported extensive prior experience using power tools. Note that every participant, regardless of experience, undershot the target during all audiohaptic trials, and overshot during auditory trials.

**Figure 4:**
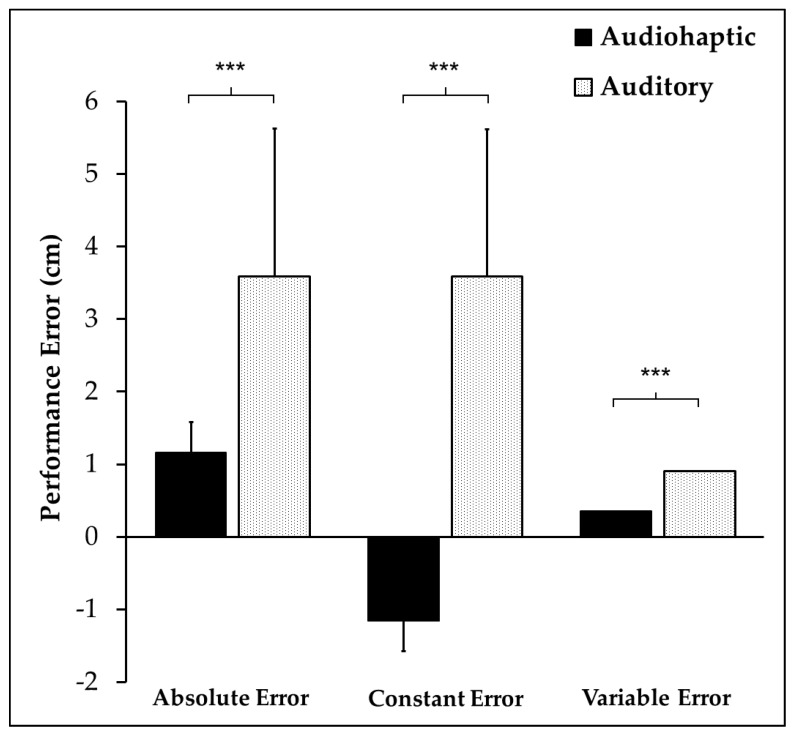
Mean and standard deviation (error bars) of performance errors in both trial types. Statistical results are noted as *** *p* < 0.001.

MANOVA results revealed a main effect of stimulus (F_(3,12)_ = 78.879, *p* < 0.001, *η*p^2^ = 0.952). Univariate testing revealed a significant stimulus by absolute error interaction (F_(1,14)_ = 17.839, *p* < 0.001, *η*p^2^ = 0.560), demonstrating larger error during the auditory trials. Significant differences were also found in the stimulus by constant error interaction (F_(1,14)_ = 92.851, *p* < 0.001, *η*p^2^ = 0.861), demonstrating negative error during the audiohaptic trials. This finding indicates that on average, participants consistently overshot (over-drilled) the target by 3.6 ± 0.9 cm during auditory trials, while undershooting by 1.2 ± 0.4 cm during the audiohaptic trials. Significant differences were also found in the stimulus by variable error interaction (F_(1,14)_ = 35.063, *p* < 0.001, *η*p^2^ = 0.715), revealing greater variability during the auditory trials. These findings indicate that participants performed better (made less errors) during the audiohaptic trials (Figure 4). There was no main effect of sex (*p* = 0.9).

### 3.2. Perceived Realness Results

**Figure 5:**
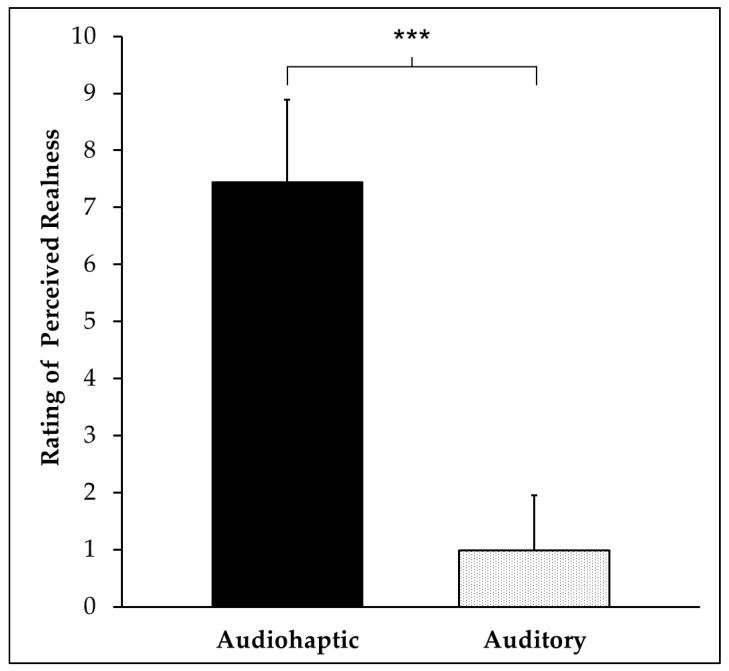
Mean and standard deviations (error bars) of perceived realness ratings in both trial types. Statistical results are noted as *** *p* < 0.001.

A paired samples *t*-test examining ratings of perceived realness revealed a significant difference between the audiohaptic and auditory-alone trial types, *t*_(14)_ = 15.858, *p* < 0.001. These results show that participants rated audiohaptic trials as being more realistic than the auditory trials (Figure 5).

## 4. Discussion

This study examined differences in motor performance between audiohaptic and auditory-only trials during a virtual drilling task. Performance errors were significantly greater in the auditory-only trials, while ratings of perceived realness were significantly higher in the audiohaptic trials. These findings support our hypothesis that audiohaptic stimuli will improve motor performance and subjective ratings of realness in a drilling simulation. These results are not surprising, as haptic feedback provides information regarding the drilling process and the physical qualities of the drilling surface.

An important strength of this study is that it provides evidence that low-fidelity haptic feedback can improve drilling performance. It is known that congruent external stimuli associated with a motor command can facilitate motor learning, and when practicing optimal behavior with these stimuli (visual, auditory, and haptic), motor skill performance can improve [4,33]. Absolute error was significantly larger in the auditory-only trials, indicating that audiohaptic stimuli leads to better performance during low-fidelity drilling tasks. This is supported by previous work on the importance of haptic perception in motor tasks [3,33]. Lin and colleagues [33] concluded that haptic feedback enhanced motor learning in a visuomotor tracking task, and these results were accompanied by differences in cortical brain activity. These findings demonstrate the importance of using task-relevant haptic feedback during motor skill acquisition.

The constant error results, which take into account the direction of error, revealed that users consistently under-drilled during audiohaptic trials. This is similar to the behavior of undershooting targets in reaching and aiming studies [39,40]. Research suggests that an initial undershoot towards a target requires less time and energy to correct when compared to overshooting [41,42]. Overshooting requires the limb to travel further and decelerate in order to return to the target, thus firing alternate muscle groups and slowing down the performance of the task [39,42]. This tendency to undershoot persists in movements with added mass and tool-use [36]. These findings have real world implications, as in many drilling tasks it is critical not to overshoot. A surgeon performing an orthopedic surgery requires precise control while drilling through bone and avoiding injury to surrounding tissues, and carpenters should abstain from drilling too deep to avoid damaging structural materials. Although this study was focused on a simulated wood-drilling task, the results can be applied to various drilling scenarios. The fact that participants were more accurate during audiohaptic trials, undershooting by an average of 1.2 cm, as compared to overshooting by 3.5 cm in the auditory trials, indicates how critical haptic sensation is for accurate motor performance. It is important to note the significant amount of undershoot during the audiohaptic trials. This is likely due to the low quality of force-feedback provided by the Falcon, and the weight of the 3D printed drill attachment that participants used to complete the trials. In a real drilling situation, the user must lift and hold the drill in place before applying force to the drilling surface. In the current study, the base of the Falcon was stationary and fixed to a tabletop, which likely affected the users’ ability to judge the drilling depth, and decreased the proprioceptive feedback from the drill-user interaction that occurs in real situations. In addition, the haptic interface was a lightweight mock drill attachment, which may not accurately represent the weight of drills used in real situations. Despite these limitations, participants performed better when exposed to low-fidelity haptic sensations, indicating that this form of haptic feedback improves accuracy.

Another strength of this study is that it indicated that even a low-fidelity device, (such as the Falcon) provided haptic information that allowed users to experience a more realistic drilling process. User experience is a fundamental aspect of simulations, whether they are used for entertainment, education, or training. This study found that participants rated the audiohaptic trials significantly higher in terms of realness when compared to the auditory-only trials, which supports our hypothesis. These results demonstrate that low-end haptic devices, when used in a relevant context, can provide enough haptic sensation to enhance ratings of reality, when compared to trials without haptic feedback. It is likely that audiohaptic interactions were able to influence a user’s experience, leading to a more realistic perception of the simulated environment. These results verify prior findings from Melaisi and colleagues [21], who found that users perceived the highest level of haptic fidelity in a drilling task when presented with a drill audio recording, compared to listening to classical music, heavy metal, white noise, or no sound at all. The same participants also felt that sound was an important aspect of their experience, which highlights the importance of contextual stimuli [21]. The current study builds on this research and indicates that low-fidelity audiohaptic stimuli can enhance a user’s perceived realism, when compared with audio stimuli alone. Future studies should assess how multimodal interactions affect task perception during a drilling simulation.

This study required participants to be seated in front of a computer simulation, which may not represent most drilling scenarios. Although seated positions are common in these types of simulations, it is possible that this protocol could have affected participants’ perception of the task. However, our results indicate large effect sizes for all statistically significant findings, so postural positioning is unlikely to have drastically influenced our results. For future simulation studies, it is recommended to examine the effects of body position on task performance and perception of multimodal stimuli.

This study provides evidence that low-fidelity haptic sensations, when combined with congruent audio stimuli, can enhance drilling performance and perceived realness. To support the findings of this research, future studies should compare the results of the simulation to those with a higher-end device or a real drill. This would provide us with more information regarding haptic fidelity and the overall perception of the simulation experience. Participants did not pull a trigger to start the drilling sensations, and this could have influenced their perception of the task. Future work should modify the task to begin when the user presses the trigger, as this would reflect a more realistic drilling process. It is also recommended that future research should explore whether variations in multimodal stimuli can influence drilling performance. Our paradigm only considered the effect of task-relevant haptic and audio stimuli on drilling performance; however it is important to understand that this task involves the interaction of other senses as well. It would be interesting to explore how a combination of visual, auditory, and haptic stimuli affect performance errors and perceived realism. This study observed the drilling behavior of participants who had prior experience operating a drill, although we did not explore differences between levels of user experience. Future work should examine how the level of user experience (i.e., novice, intermediate, expert) affects drilling performance and overall perception of the simulated task. Although we found no sex differences in performance, we had a small sample of participants in each group. As differences between sexes might be expected in these types of studies, it is recommended that future work should examine these differences with a larger sample size. Motor learning also involves skill retention and transfer, which we did not test for in this study. Manipulating the experiment to create a more difficult task, and adding a second session the following day to assess skill retention could provide us with critical information on how humans learn psychomotor skills in simulations. It is also possible that drilling to shallow depths provides more difficulty for participants, in which case future studies could explore the effects of altering the target depth.

## Supplementary Materials

The following are available online at https://www.mdpi.com/2076-3425/10/1/21/s1, Table S1: Participant data for audiohaptic trials; Table S2: Participant data for auditory trials.

## References

[1] Siu K.-C., Best B.J., Kim J.W., Oleynikov D., Ritter F.E. (2016). Adaptive virtual reality training to optimize military medical skills acquisition and retention. Milit. Med..

[2] Williams-Bell F.M., Kapralos B., Hogue A., Murphy B., Weckman E. (2015). Using serious games and virtual simulation for training in the fire service: A review. Fire Technol..

[3] Coles T., Meglan D., John N.W. (2011). The role of haptics in medical training simulators: A survey of the state of the art. IEEE Trans. Haptics.

[4] Maxwell J., Masters R., Kerr E., Weedon E. (2001). The implicit benefit of learning without errors. Q. J. Exp. Psychol. Sec. A.

[5] Cox D.J., Davis M., Singh H., Barbour B., Nidiffer F.D., Trudel T., Mourant R., Moncrief R. (2010). Driving rehabilitation for military personnel recovering from traumatic brain injury using virtual reality driving simulation: A feasibility study. Milit. Med..

[6] Brydges R., Carnahan H., Backstein D., Dubrowski A. (2007). Application of motor learning principles to complex surgical tasks: Searching for the optimal practice schedule. J. Motor Behav..

[7] Flavián C., Ibáñez-Sánchez S., Orús C. (2019). The impact of virtual, augmented and mixed reality technologies on the customer experience. J. Bus. Res..

[8] Seitz A.R., Kim R., van Wassenhove V., Shams L. (2007). Simultaneous and independent acquisition of multisensory and unisensory associations. Perception.

[9] Srinivasan M.A. (2016). What Is Haptics?.

[10] Culbertson H., Schorr S.B., Okamura A.M. (2018). Haptics: The present and future of artificial touch sensation. Annu. Rev. Control Robot. Auton. Syst..

[11] Melaisi M., Rojas D., Kapralos B., Uribe-Quevedo A., Collins K. (2018). Multimodal Interaction of Contextual and Non-Contextual Sound and Haptics in Virtual Simulations. Informatics.

[12] Coles T., John N.W., Gould D.A., Caldwell D.G. Haptic palpation for the femoral pulse in virtual interventional radiology. Proceedings of the 2009 Second International Conferences on Advances in Computer-Human Interactions.

[13] Brydges R., Carnahan H., Rose D., Rose L., Dubrowski A. (2010). Coordinating progressive levels of simulation fidelity to maximize educational benefit. Acad. Med..

[14] Basdogan C., De S., Kim J., Muniyandi M., Kim H., Srinivasan M.A. (2004). Haptics in minimally invasive surgical simulation and training. IEEE Comput. Graph. Appl..

[15] McCracken H.S., Murphy B.A., Glazebrook C.M., Burkitt J.J., Karellas A.M., Yielder P.C. (2019). Audiovisual multisensory integration and evoked potentials in young adults with and without Attention-Deficit/Hyperactivity Disorder. Front. Hum. Neurosci..

[16] Maravita A., Spence C., Driver J. (2003). Multisensory integration and the body schema: Close to hand and within reach. Curr. Biol..

[17] Roll J.P., Roll R., Velay J.-L. (1991). Proprioception as a link between body space. Brain and Space.

[18] Gallagher S. (1986). Body image and body schema: A conceptual clarification. J. Mind Behav..

[19] Iriki A., Tanaka M., Iwamura Y. (1996). Coding of modified body schema during tool use by macaque postcentral neurones. Neuroreport.

[20] Maravita A., Clarke K., Husain M., Driver J. (2002). Active tool use with the contralesional hand can reduce cross-modal extinction of touch on that hand. Neurocase.

[21] Melaisi M., Nguyen M., Uribe A., Kapralos B. The effect of sound on haptic fidelity perception. Proceedings of the 2017 IEEE Global Engineering Education Conference (EDUCON).

[22] Parsons T.D. (2015). Virtual reality for enhanced ecological validity and experimental control in the clinical, affective and social neurosciences. Front. Hum. Neurosci..

[23] Laurienti P.J., Kraft R.A., Maldjian J.A., Burdette J.H., Wallace M.T. (2004). Semantic congruence is a critical factor in multisensory behavioral performance. Exp. Brain Res..

[24] Hébert S., Béland R., Dionne-Fournelle O., Crête M., Lupien S.J. (2005). Physiological stress response to video-game playing: The contribution of built-in music. Life Sci..

[25] Lipscomb S.D., Zehnder S.M. (2004). Immersion in the virtual environment: The effect of a musical score on the video gaming experience. J. Physiol. Anthropol. Appl. Hum. Sci..

[26] Sanchez-Vives M.V., Slater M. (2005). From presence to consciousness through virtual reality. Nat. Rev. Neurosci..

[27] Witmer B.G., Singer M.J. (1998). Measuring presence in virtual environments: A presence questionnaire. Presence Teleoper. Virtual Environ..

[28] Baumgartner T., Valko L., Esslen M., Jäncke L. (2006). Neural correlate of spatial presence in an arousing and noninteractive virtual reality: An EEG and psychophysiology study. CyberPsychol. Behav..

[29] Gregory R. (1967). Origin of eyes and brains. Nature.

[30] Posner M.I., Nissen M.J., Klein R.M. (1976). Visual dominance: An information-processing account of its origins and significance. Psychol. Rev..

[31] Hecht D., Reiner M. (2009). Sensory dominance in combinations of audio, visual and haptic stimuli. Exp. Brain Res..

[32] Hartcher-O’Brien J., Gallace A., Krings B., Koppen C., Spence C. (2008). When vision ‘extinguishes’ touch in neurologically-normal people: Extending the Colavita visual dominance effect. Exp. Brain Res..

[33] Lin C.-L., Shaw F.-Z., Young K.-Y., Lin C.-T., Jung T.-P. (2012). EEG correlates of haptic feedback in a visuomotor tracking task. NeuroImage.

[34] Faul F., Erdfelder E., Lang A.-G., Buchner A. (2007). G*Power 3: A flexible statistical power analysis program for the social, behavioral, and biomedical sciences. Behav. Res. Methods.

[35] Knox J.J., Hodges P.W. (2005). Changes in head and neck position affect elbow joint position sense. Exp. Brain Res..

[36] Burkitt J.J., Staite V., Yeung A., Elliott D., Lyons J.L. (2015). Effector mass and trajectory optimization in the online regulation of goal-directed movement. Exp. Brain Res..

[37] Guth D. (1990). Space Saving Statistics: An Introduction to Constant Error, Variable Error, and Absolute Error. Peabody J. Educ..

[38] Richardson J.T. (2011). Eta squared and partial eta squared as measures of effect size in educational research. Educ. Res. Rev..

[39] Engelbrecht S.E., Berthier N.E., O’Sullivan L.P. (2003). The Undershoot Bias: Learning to Act Optimally under Uncertainty. Psychol. Sci..

[40] Roberts J.W., Burkitt J.J., Elliott D., Lyons J.L. (2016). The impact of strategic trajectory optimization on illusory target biases during goal-directed aiming. J. Motor Behav..

[41] Lyons J., Hansen S., Hurding S., Elliott D. (2006). Optimizing rapid aiming behaviour: Movement kinematics depend on the cost of corrective modifications. Exp. Brain Res..

[42] Elliott D., Hansen S., Mendoza J., Tremblay L. (2004). Learning to optimize speed, accuracy, and energy expenditure: A framework for understanding speed-accuracy relations in goal-directed aiming. J. Motor Behav..

