# Supplementary material for: Audiohaptic Feedback Enhances Motor Performance in a Low-Fidelity Simulated Drilling Task
Source: Brain Sci. 2019 Dec 31;10(1):21. doi: 10.3390/brainsci10010021 (PMC7016775; doi:10.3390/brainsci10010021)
Supplement: Supplementary file 1 [file brainsci-10-00021-s001.zip › Supplementary Data/Supplementary Table 1.docx]

Audiohaptic feedback enhances motor performance in a low-fidelity simulated drilling task

| Supplementary Table 1. Participant data for audiohaptic trials | | | | | |
| --- | --- | --- | --- | --- | --- |
| Participant | Drilled Depth (cm) | Absolute Error (cm) | Constant Error (cm) | Variable Error (cm) | Realness Rating |
| 1 | 0.48 | 1.52 | -1.52 | 0.22 | 8.88 |
| 2 | 0.46 | 1.53 | -1.54 | 0.13 | 9.95 |
| 3 | 0.61 | 1.39 | -1.39 | 0.49 | 7.75 |
| 4 | 1.88 | 0.12 | -0.12 | 0.56 | 8.34 |
| 5 | 1.36 | 0.64 | -0.64 | 0.39 | 8.72 |
| 6 | 0.27 | 1.73 | -1.73 | 0.16 | 7.21 |
| 7 | 1.00 | 1.00 | -1.00 | 0.80 | 7.46 |
| 8 | 0.42 | 1.58 | -1.58 | 0.18 | 8.02 |
| 9 | 1.07 | 0.93 | -0.93 | 0.61 | 6.45 |
| 10 | 1.03 | 0.97 | -0.97 | 0.25 | 4.51 |
| 11 | 1.11 | 0.89 | -0.89 | 0.34 | 7.86 |
| 12 | 0.76 | 1.24 | -1.24 | 0.26 | 6.38 |
| 13 | 0.83 | 1.17 | -1.17 | 0.35 | 7.40 |
| 14 | 0.46 | 1.54 | -1.54 | 0.21 | 7.91 |
| 15 | 0.89 | 1.11 | -1.11 | 0.33 | 4.84 |
