# Supplementary material for: Audiohaptic Feedback Enhances Motor Performance in a Low-Fidelity Simulated Drilling Task
Source: Brain Sci. 2019 Dec 31;10(1):21. doi: 10.3390/brainsci10010021 (PMC7016775; doi:10.3390/brainsci10010021)
Supplement: Supplementary file 1 [file brainsci-10-00021-s001.zip › Supplementary Data/Supplementary Table 2.docx]

Audiohaptic feedback enhances motor performance in a low-fidelity simulated drilling task

| Supplementary Table 2. Participant data for auditory trials | | | | | |
| --- | --- | --- | --- | --- | --- |
| Participant | Drilled Depth (cm) | Absolute Error (cm) | Constant Error (cm) | Variable Error (cm) | Realness Rating |
| 1 | 4.99 | 2.99 | 2.99 | 0.94 | 0.76 |
| 2 | 6.90 | 4.93 | 4.90 | 0.83 | 0.20 |
| 3 | 7.65 | 5.65 | 5.65 | 0.68 | 0.23 |
| 4 | 7.46 | 5.46 | 5.46 | 0.58 | 1.52 |
| 5 | 7.69 | 5.69 | 5.69 | 0.70 | 3.01 |
| 6 | 3.60 | 1.60 | 1.60 | 0.65 | 0.53 |
| 7 | 6.47 | 4.47 | 4.47 | 1.27 | 0.86 |
| 8 | 4.98 | 2.98 | 2.98 | 1.47 | 0.51 |
| 9 | 3.61 | 1.61 | 1.61 | 0.96 | 2.65 |
| 10 | 7.83 | 5.83 | 5.83 | 0.50 | 0.01 |
| 11 | 6.38 | 4.38 | 4.38 | 1.51 | 0.82 |
| 12 | 7.87 | 5.87 | 5.87 | 0.54 | 1.80 |
| 13 | 2.83 | 0.83 | 0.83 | 1.28 | 0.00 |
| 14 | 3.04 | 1.04 | 1.04 | 0.73 | 1.79 |
| 15 | 2.52 | 0.52 | 0.52 | 0.86 | 0.00 |
